# Certificateless aggregate signcryption scheme with multi-ciphertext equality test for the internet of vehicles

**DOI:** 10.1371/journal.pone.0322185

**Published:** 2025-05-27

**Authors:** Xiaodong Yang, Xilai Luo, Ruixia Liu, Songyu Li, Ke Yao

**Affiliations:** College of Computer Science and Engineering, Northwest Normal University, Lanzhou, China; University of Electronic Science and Technology of China, CHINA

## Abstract

The Internet of Vehicles (IoV) facilitates connectivity among vehicles, roadside units, and smart terminals, enabling the evolution of traditional traffic networks into intelligent transport systems. The IoV has an open communication character, which enables various applications and services. However, this also exposes it to the risk of message tampering or the leaking of private data in the communication process. Such vulnerabilities may lead to security issues. At present, there are many solutions to solve the above problems, but most of them have great computational and communication overhead. Multi-ciphertext equality test can compare the equality between two ciphertexts without decryption, which avoids the user ’s repeated decryption of the same ciphertext to a certain extent. However, it still has a large computational overhead. For the above problems, we propose a certificateless aggregate signcryption scheme for the IoV that supports multi-ciphertext equality testing.

The proposed scheme addresses the key escrow and certificate management issues inherent in identity-based systems by employing a certificateless signcryption mechanism. To prevent redundant retrieval of ciphertexts that correspond to identical plaintexts, a multi-ciphertext equivalence test feature has been incorporated. Furthermore, the aggregation capability of this scheme significantly enhances the efficiency of signing multiple vehicle data entries. By leveraging the computational complexities associated with the Diffie-Hellman problem and the discrete logarithm problem, it is demonstrated that the scheme maintains confidentiality and unforgeability within the random oracle model. When compared to similar schemes, this approach exhibits reduced computational overhead while providing superior security features.

## Introduction

The Internet of Thing, which connects various devices and sensors through the IoV data acquisition and exchange. Under the influence of Internet of things technology, people ’s daily life has become more convenient, such as smart home, medical system, vehicle monitoring and environmental perception [1].

The rapid advancement in wireless network communication technology has heightened the demand for more user-centric transportation services [2]. This has led to the development of the IoV, which leverages vehicles as primary carriers to enable smart interactions among vehicles, roads, infrastructure, and networks. Connected vehicles can share vital information such as driving statuses, speeds, road conditions, locations, and incidents. This capability allows the IoV to effectively manage traffic flow, reducing congestion and accidents, and thereby enhancing travel safety and comfort for drivers and passengers [3,4]. However, the IoV also encounters security and privacy challenges due to network instability and complex information transmission, including risks like malicious eavesdropping [5–7].

Maintaining network connectivity in wireless communication networks is crucial. To address communication bottlenecks and enhance connectivity, effective node deployment is essential [8,9]. For improved traffic management, the IoV system can significantly reduce congestion and accidents through real-time communication and coordination between vehicles and Road Side Units (RSUs). However, this system also raises security and privacy concerns due to the collection and exchange of sensitive data, such as vehicle locations and speeds [11,12]. As vehicles, which act as primary information carriers, move rapidly, their frequent position changes necessitate switching between different network nodes. This scenario introduces risks from malicious attacks using forged nodes, potentially compromising system integrity and driver safety, and possibly causing traffic accidents [13–15].

To address the security challenges in information transmission within the IoV, aggregate signcryption technology has been developed. This technology combines aggregation techniques with signcryption systems to perform both digital encryption and digital signature in a single logical step [16]. It enables the merging of multiple signcryption messages into one, allowing the recipient to simultaneously verify several ciphertexts, which reduces the time needed for verification. Additionally, aggregated ciphertext occupies less space compared to multiple individual ciphertexts, thereby lowering network bandwidth and computational overhead requirements. By allowing multiple data sources or vehicles to collectively signcrypt information, aggregate signcryption enhances security, privacy, and authenticity. Integrating this technology into the IoV can thus greatly advance the intelligent development of transportation systems and improve safety performance [17].

The proposed scheme primarily contributes in the following ways:

1) The use of certificateless technology solves the problem of certificate management and key escrow, thereby ensuring the confidentiality and unforgeability of vehicle data in the IoV.
2) By using aggregate signcryption technology, aggregators can signcrypte multiple data, which improves the signcryption efficiency in multi-user environment.
3) The proposed scheme supports multi-ciphertext equality test. Testers can use the test trapdoor uploaded by data users to match multiple ciphertexts simultaneously, which enables multi-user retrieval and multi-ciphertext equality test, and reduces the computational cost of the equality test process in a multi-user environment.
4) Our scheme uses aggregate signcryption technology and multi-ciphertext equality test to solve the problem of high computational overhead and low computational efficiency in signcryption schemes, while improving the security of data.

We compare and analyze similar schemes in terms of functional characteristics and computational cost, and the results indicate that the proposed scheme has lower computational cost and higher security.

## Related work

With the rapid development of the IoV, more and more vehicles and devices are integrated into it and constitute a huge network. The focus in this network is data security and privacy protection. The traditional encryption algorithms in IoV are limited by factors such as computation and storage resources, the complexity of certificate management and revocation. Therefore, certificateless encryption technology has become an effective means to address these issues.

To ensure the security of data acquisition, processing and access in the IoV, many scholars at home and abroad have proposed to add encryption mechanism to the IoV. From the perspective of security requirements, data authenticity and confidentiality are two emphases in many such applications [18].

Cryptography provides a solution to these security requirements, and has carried out many research work. The concept of signcryption and specific signcryption schemes was first proposed by Zheng [19] in 1997. In 2002, Baek *et al* [20] developed a security model for the signcryption scheme and demonstrated the security of Zheng’s scheme. In 2008, Barbosa *et al*. [21] introduced a certificateless signcryption scheme and presented a specific security model that combined the benefits of certificateless cryptography. Subsequently, several certificateless signcryption schemes have been proposed [22].

Mei *et al*. [23] proposed an efficient certificateless scheme with conditional privacy, which greatly reduced the computational overhead by using aggregation technology. Kasyoka *et al*. [24] proposed a certificateless signcryption scheme without bilinear pairing, which significantly reduces computational overhead. Dohare *et al*. [25] proposed a new certificateless aggregate signcryption scheme (CLASS). Compared to the traditional certificate-based public key cryptosystem (CPKC) scheme, this scheme does not require the distribution of certificates in advance, to reduce complexity and overhead.

Ullah *et al*. [26] proposed a new anonymous certificateless signcryption scheme that supports aggregate signcryption operations for multiple users, but uses computationally expensive bilinear pairing operations. However, this scheme cannot effectively retrieve ciphertext, which leads to the high cost of RSUs screening vehicle data.

In the IoV, multi-ciphertext matching is often required. However, traditional ciphertext equality test techniques can only compare the equality between two ciphertexts. For IoV, which with huge data, there are many ciphertexts waiting for equality test. Therefore, we need to divide multi-ciphertext into many groups to compare them in pairs and perform equality test separately. This obviously does not meet the needs of IoV, a resource-constrained network. The huge equality test will bring a greater burden to the system, thereby reducing the utilization of data. To enhance the computational efficiency of ciphertext equality test in multi-ciphertext scenarios, Susilo proposed a public-key encryption with multi-ciphertext equality test (PKE-MET). Although the scheme supports the equality test of more than two ciphertexts, there are some problems such as high cost for certificate management.

To address the aforementioned issues, we propose the Certificateless Aggregate Signcryption Scheme with Multi-Ciphertext Equality Test for the Internet of Vehicles presents a certificateless aggregate signcryption scheme that facilitates multi-ciphertext equality test. The scheme provides confidentiality, authenticity and unforgeability, and resists type I and type II adversarys with low computational cost. The proposed signcryption scheme significantly enhances computational efficiency when compared to existing schemes.

## Preliminaries

### Mathematical assumption

Discrete Logarithm Problem (DLP): Given two large prime numbers *p* and *q* that satisfy the condition p|q−1, the generator of group $Z_{p}^{*}$ is *P*, given tuple $(P,aP)\in Z_{p}^{*}$, calculate *a*.

Computational Diffie–Hellman (CDH) Problem: Given two large prime numbers *p* and *q* that satisfy the condition p|q−1, the generator of group $Z_{p}^{*}$ is *P*, and given tuple $(P,aP,bP)\in Z_{p}^{*}$, calculate *abP*.

### Security model

The proposed scheme in this paper satisfies the confidentiality of messages and the unforgeability of ciphertexts, namely the indistinguishability under adaptive chosen ciphertext attacks (IND-CCA2) and the existential unforgeability under adaptive chosen message attacks (EUF-CMA).

To demonstrate the security of the scheme, we define two types of adversaries.

1) Type I adversary: One type of adversary is not permitted to access the master key *s*, but can replace the public key of a receiver. In addition, it cannot determine the ciphertext that is calculated by which message with the absence of trapdoor. This model is considered as an IND-CCA2 security model.
2) Type II adversary: Another type of adversary is allowed to obtain the master key, but is unable to replace the public key of a receiver. In addition, it cannot determine the ciphertext that is calculated by which message with the absence of trapdoor. This model is also considered as an IND-CCA2 security model.

**Definition 1 (IND-CCA2-1).** If no adversary ${𝒜}_{1}$ wins the following game by a non-negligible margin in bounded polynomial time, this signcryption scheme is IND-CCA2 secure. The adversary ${𝒜}_{1}$ interacts with the challenger 𝒞 by the following steps:

1. **Setup:** 𝒞 executes system initialization algorithm, outputs system parameters *para* and master key *s*, returns *para* to ${𝒜}_{1}$, and keeps *s* in secret. The system initialization algorithm is executed by 𝒞, which outputs the system parameters *para* and master key *s*. The algorithm then returns *para* to ${𝒜}_{1}$ and keeps the master key *s* secretly.
2. **Phase 1:** ${𝒜}_{1}$ performs the following polynomial time queries of finite order in an adaptive manner:
  1) Signcryption query: $\left(ID_{i},m_{i},ID_{j}\right)$ is provided to 𝒞 by ${𝒜}_{1}$. 𝒞 execute the signcryption algorithm to obtain the ciphertext *C*_*i*_ and return it to ${𝒜}_{1}$.
  2) Unsigncryption query: ${𝒜}_{1}$ submits $\left(ID_{i},C_{i},ID_{j}\right)$ to 𝒞 for validation. Upon verifying the validity of the ciphertext, 𝒞 proceeds with the unsigncryption algorithm to recover plaintext message *m*_*i*_, which it then returns to ${𝒜}_{1}$. If the ciphertext is found invalid, 𝒞 returns $\prime {⊥}^{\prime }$.
3. **Challenge:** ${𝒜}_{1}$ chooses the challenge identity $\left(ID_{i},ID_{j}\right)$ and two plaintext messages $\left(m_{0},m_{1}\right)$ of identical length. ${𝒜}_{1}$ unables to query the private key of the challenge identity. 𝒞 randomly selects ξ∈{0,1}, calculates $m_{\xi }$ ciphertexts ${\text{C}}^{*}$ and returns the corresponding result to ${𝒜}_{1}$.
4. **Phase 2:** After obtaining ${\text{C}}^{*}$, ${𝒜}_{1}$ continues to execute the associated query in **Phase 1**. $A_{1}$ cannot query the private key of identity $I{D_{j}}^{*}$, nor can it perform an unsigncryption query on ${\text{C}}^{*}$.
5. **Guess:** ${𝒜}_{1}$ exports a guess value of $\xi^{*}\in \{0,1\}$, if $\xi^{*}=\xi$, ${𝒜}_{1}$ wins in Game 1.

**Definition 2 (IND-CCA2-2).** If no adversary ${𝒜}_{2}$ wins the following game by a non-negligible margin in bounded polynomial time, this signcryption scheme is IND- CCA2 secure. The adversary ${𝒜}_{2}$ interacts with the challenger 𝒞 by the following steps:

1. **Setup: **𝒞 selects challenge identity *ID*_*j*_, executes initialization algorithm, randomly selects $a\in Z_{q}^{*}$, calculates system public key *P*_*pub*_ = *aP*, and returns system parameters $para=\{p,q,P,P_{pub},H_{0},H_{1},H_{2},H_{3},H_{4},H_{5}\}$ and *ID*_*j*_ to ${𝒜}_{2}$.
2. **Phase 1:** Similar to theorem 1, the difference is that public key replacement query and partial private key query cannot be performed.
3. **Phase 2:** Similar to theorem 1, the difference is that the secret value query of *ID*_*j*_ cannot be performed.

The challenge phase and the guess phase are the same as theorem 1. In the end, the adversary ${𝒜}_{2}$ wins the game.

**Definition 3 (EUF-CMA-1).** If adversary ${𝒜}_{1}$ is malicious users who does not possess the system’s master key, but can replace any user’s public key. If scheme can resist adaptive chosen messages attacks from ${𝒜}_{1}$, then the scheme satisfies the existential unforgeability under adaptive chosen messages attack (EUF-CMA).

1. **Setup: **𝒞 sets *P*_*pub*_ = *aP*, and returns system parameters $para=\{p,q,P,P_{\text{pub}},$$H_{0},H_{1},H_{2},H_{3},H_{4},H_{5}\}$ to ${𝒜}_{1}$.
2. **Queries phases: **${𝒜}_{1}$ adaptively performs hash query, public key query, private key query, public key replacement query signcryption query and unsigncryption query.
3. **Forgery phase:** In the forgery phase, ${𝒜}_{1}$ attempts to construct a fake message or signature and submits it to the signcryption scheme for verification through an interactive process. If the signcryption scheme can accurately identify this forgery and refuse to accept false messages or signatures, then the scheme satisfies EUF- CMA. After the queries phases, forge and output *n* signers $ID_{i}^{*}$ to create an aggregate signature ciphertext $\sigma_{agg}^{*}=\left({\left\{C_{i}^{*}\right\}}_{i=1,2,\cdots ,n},X_{agg}^{*}\right)$for the message $m_{i}^{*}$. Assuming that at least one out of the *n*users, specifically user *D*_1_, has not been queried for their secret value. At the same time, *D*_1_ has not conducted aggregate signcryption query, and $\sigma_{agg}^{*}$ is a valid ciphertext, then ${𝒜}_{1}$ wins the game.

**Definition 4 (EUF-CMA-2).** If adversary ${𝒜}_{2}$ is an honest but curious Key Generation Center (KGC) who can obtain the system’s master key, but cannot replace users’ public keys. If scheme can resist adaptive chosen messages attacks from ${𝒜}_{2}$, then the scheme satisfies the EUF-CMA.

1. **Setup: **𝒞 sets *P*_*pub*_ = *aP*, and returns system parameters $para=\{p,q,P,P_{\text{pub}},$$H_{0},H_{1},H_{2},H_{3},H_{4},H_{5}\}$ and system master key *s* to ${𝒜}_{2}$.
2. **Queries phases: **${𝒜}_{2}$ owns the system master key *s*. ${𝒜}_{2}$ can adaptively perform hash query, public key query, private key query, signcryption query and unsigncryption query.
3. **Forgery phase:** In the forgery phase, ${𝒜}_{2}$ attempts to construct a fake message or signature and submits it to the signcryption scheme for verification through an interactive process. If the signcryption scheme can accurately identify this forgery and refuse to accept false messages or signatures, then the scheme satisfies EUF- CMA. After the queries phases, forge and output *n* signers $ID_{i}^{*}$ to create an aggregate signature ciphertext $\sigma_{agg}^{*}=\left({\left\{C_{i}^{*}\right\}}_{i=1,2,\cdots ,n},X_{agg}^{*}\right)$for the message $m_{i}^{*}$. Assuming that at least one out of the *n*users, specifically user *D*_1_, has not been queried for their secret value. At the same time, *D*_1_ has not conducted aggregate signcryption query, and $\sigma_{agg}^{*}$ is a valid ciphertext, then ${𝒜}_{2}$ wins the game.

## Scheme construction

In the IoV, when urban traffic is congested, a RSU typically needs to manage hundreds of vehicles, and can use aggregate signcryption technology to batch verify signcryption information from a large number of On- Board Units (OBUs) in a short time. The scheme is mainly composed of the following entities.

1) **OBU :** The vehicle data owner, it signs the message and sends it to the RSU.
2) **RSU :** The vehicle data receiver, decrypts and verifies the received ciphertext, signs and broadcasts the traffic information.
3) **KGC :** Initialize the system, generate the system master key and system parameters.
4) **Aggregate Signcryption Generator (Agg-tor) :** Aggregate the ciphertext from multiple vehicles and upload the aggregated ciphertext to cloud server.
5) **Tester :** Perform the equality test algorithm on multiple vehicle ciphertexts and return the coorsponding results to cloud server.
6) **Cloud Service Provider (CSP) :** Cloud Service Provider, provides flexible storage capacity and efficient data transmission.

**Fig 1:**
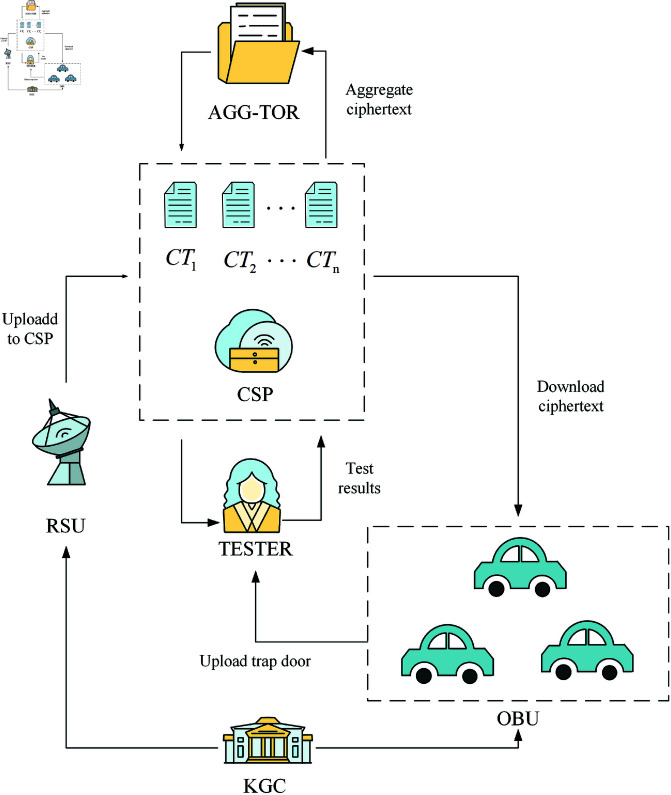
System model.

The system model is indicated in Fig 1.

We propose a certificateless aggregate signcryption scheme that does not require bilinear pairs and supports equality test. The specific implementation process, tailored to the application scenario of the IoV is as follows:

1. **Setup:** Given the security parameter *k*, we generate two large prime numbers *p* and *q* that satisfy p∣(q − 1). Let *G* be a cyclic group on an elliptic curve. In group *G*, *P* is a generator of order *q*. The Key Generation Center (KGC) defines hash functions:
  $H_{0}:\{0,1{\}}^{*}\to$ $Z_{q}^{*}$, $H_{1}:\{0,1{\}}^{*}\times G^{2}\to$ $Z_{q}^{*}$, *H*_2_: $Z_{q}^{*}$ $\times \{0,1{\}}^{*}\times G^{4}\times$ $Z_{q}^{*}$ $\times G^{3}$ $\times Z_{q}^{*}\to \{0,1{\}}^{*}$, $H_{3}:\{0,1{\}}^{*}\to Z_{q}^{*}$, $H_{4}:G\to \{0,1{\}}^{2l}$, $H_{5}:\{0,1{\}}^{*}\to \{0,1{\}}^{l}$.
  The master key s∈ $Z_{q}^{*}$ is randomly selected and kept secret by the KGC. The system’s public key is computed as *P*_*pub*_ = *sP*, and the system parameters are published as $para=\{p,q,P,P_{pub},H_{0},H_{1},H_{2},H_{3},H_{4},H_{5}\}$.
2. **Key generation:** There is the process for key selection and partial private key extraction:
  1) The vehicle mounted unit *OBU*_*i*_ randomly selects $x_{i}\in {𝑍}_{\text{q}}^{*}$ as its secret value and sets $X_{i}=x_{i}P$, and $ID_{i}\in \{0,1{\}}^{*}$, then sending $\left(ID_{i},X_{i}\right)$ to the KGC.
  2) KGC randomly selects $r_{i}\in {𝑍}_{\text{q}}^{*}$, calculates $d_{i}=r_{i}+sH_{0}(ID_{i},PK_{i})$ and $M_{i}=r_{i}P$, and sends $\left(d_{i},M_{i}\right)$ to *OBU*_*i*_.
  3) To ensure the validity of the partial public key and the partial private key, *OBU*_*i*_ receives $\left(d_{i},M_{i}\right)$ sent by KGC and verifies whether equation *d*_*i*_*P* =  $M_{i}+P_{pub}H_{0}(ID_{i})$ is true. If the equation is true, OBUI calculates part of the private key $D_{i}=sd_{i}$.
  4) OBU calculates $PK_{i,1}=(X_{i},M_{i})$, $SK_{i,1}=(D_{i},x_{i})$, $SK_{i,2}=s\cdot H_{1}(ID_{i},$ *PK*_*i*,1_), $PK_{i,2}=SK_{i,2}P$, $PK_{i,3}=H_{1}(ID_{i},PK_{i,1})$, $SK_{i,3}=x_{i}+sPK_{i,3}$.
  5) OBU returns public key $PK_{i}=(PK_{i,1},PK_{i,2},PK_{i,3})$ and private key *SK*_*i*_ $=(SK_{i,1},SK_{i,2},SK_{i,3})$ to *OBU*_*i*_.
    Similarly, the public key of *RSU*_*j*_ is $PK_{j}=(PK_{j,1},PK_{j,2},PK_{j,3})$, and the private key is $SK_{j}=(SK_{j,1},SK_{j,2},SK_{j,3})$.
3. **Signcryption:** Assuming the identity of *OBU*_*i*_ participating in the signcryption is *ID*_*i*_, the identity of the aggregate signcryption resever *RSU*_*j*_ is *ID*_*j*_ with a public key of *PK*_*j*_, and the message $m_{i}(1\leq i\leq n)$, *OBU*_*i*_ performs the following algorithm to sign the plaintext message *m*_*i*_:
  1) Randomly select $a_{i},b_{i},t_{i}\in Z_{q}^{*}$ and calculate $T_{i}=t_{i}P$, $C_{i,1}=a_{i}P$, *C*_*i*,2_ =  *b*_*i*_*P*, $R_{i}=t_{i}P_{pub}H_{1}(ID_{i},PK_{i})$.
  2) Calculate $h_{i}=H_{2}(m_{i},ID_{i},ID_{j},T_{i},PK_{i},PK_{j})$, $V_{i}=d_{i}\text{+}h_{i}a_{i}+t_{i}h_{i}$, $C_{i,3}=V_{i}P$, $C_{i,4}=R_{i}⊕(m_{i}∥V_{i})$.
  3) Calculate $f_{i,0}=H_{3}(m_{i},n)$, $f_{i,1}=H_{3}(m_{i},n,f_{i,0}),\cdots ,f_{i,n-1}=H_{3}(m_{i},n,$ $f_{i,0},\cdots ,f_{i,n-1})$, where $f(N_{i})=f_{i,0}+f_{i,1}N_{i}+f_{i,2}N_{i}^{2}+\cdots +f_{i,n-1}N_{i}^{n-1}$.
  4) Calculate $C_{i,5}=H_{4}(t_{i}X_{i}+R_{i})⊕\left(N_{i}\|f(N_{i})\right)$, $C_{i,6}=H_{5}(n\|C_{i,1}\|\cdots \|C_{i,5}$ $\left\|b_{i}PK_{j,2}\|f_{i,0}\|\cdots \|f_{i,n-1}\right)$.
  5) Upload ciphertext $C_{i}=(u_{i},C_{i,1},C_{i,2},C_{i,3},C_{i,4},C_{i,5},C_{i,6})$ to cloud storage, of which *u*_*i*_ = *n*.
4. **Multi-Ciphertext Equality Test:** At this segment, *OBU*_*i*_ decrypts the ciphertext *C*_*i*_ sent by *RSU*_*j*_ to obtain a plaintext message *m*_*i*_, with the specific steps as follows:
  The *n* RSUs send equivalent test trapdoors $tk_{j}=SK_{j,3}$ to the tester, of which j∈{1,2,⋯,n}. The tester downloads *n* ciphertexts $C_{1},C_{2},\cdots ,C_{n}$ that the vehicle wants to test from the cloud server, and performs the following multi- ciphertext equality test operations:
  - Check whether $u_{1}=u_{2}=\cdots =u_{n}=n$ is valid. If it is, the tester continues to perform the following operations. Otherwise, terminate the operation and output $\prime {⊥}^{\prime }$.
  - The tester calculates $N_{i}∥f(N_{i})=C_{i,5}⊕H_{4}(T_{i}\cdot tk_{j})$ and obtain $f(N_{i})=f_{i,0}+f_{i,1}N_{i}+f_{i,2}N_{i}^{2}+\cdots +f_{i,n-1}N_{i}^{n-1}$ by the signature secret algorithm. The tester combines the *n* equations into the following system of equations:
    $$\left\{\begin{array}{c} f(N_{1})=f_{1,0}+f_{1,1}N_{1}+f_{1,2}N_{1}^{2}+\cdots +f_{1,n-1}N_{1}^{n-1}\\ f(N_{2})=f_{2,0}+f_{2,1}N_{2}+f_{2,2}N_{2}^{2}+\cdots +f_{2,n-1}N_{2}^{n-1}\\ \vdots \\ f(N_{n})=f_{n,0}+f_{n,1}N_{n}+f_{n,2}N_{n}^{2}+\cdots +f_{n,n-1}N_{n}^{n-1} \end{array}\right.$$ (1)
  - Check whether equation $C_{i,6}=H_{5}(n,C_{i,1},\ldots ,C_{i,5},T_{i}\cdot k_{j},f_{i,0},\ldots ,f_{i,n-1})$ holds. If it holds, the tester outputs a test consequence of $\prime 1^{\prime }$ to the cloud server. Otherwise, the test consequence output to the cloud server is $\prime 0^{\prime }$.
5. **Aggregate signcryption:**
  1) Calculate $X_{agg}=\sum_{i=1}^{n}C_{i,3}$.
  2) The aggregated ciphertext $\sigma_{agg}=\left({\left\{C_{i}\right\}}_{i=1,2,\cdots ,n},X_{agg}\right)$ is uploaded to the cloud server for storage.
6. **Aggregate unsigncryption:**
  1) Calculate $R_{i}^{\prime }=D_{j}C_{i,1}$, $m_{i}^{\prime }∥V_{i}^{\prime }\text{=}C_{i,4}⊕R_{i}^{\prime }$.
  2) Calculate $f_{i,0}^{\prime },f_{i,1}^{\prime },\cdots ,f_{i,n}^{\prime },N_{i}^{\prime }∥f(N_{i}^{\prime })=C_{i,5}⊕H_{4}(T_{i}SK_{j,3})$ based on $m_{i}^{\prime }$.
  3) Calculate $X_{agg}^{\prime }=\sum_{i=1}^{n}V_{i}^{\prime }P$, and $X_{agg}^{*}=\sum_{i=1}^{n}(M_{i}\;+\;P_{pub}H_{1}(ID_{i},PK_{i})\;+\;h_{i}^{\prime }C_{i,1}\;+\;h_{i}^{\prime \prime }T_{i})$.
  4) Check whether equations $X_{agg}^{*}=X_{agg}^{\prime }$ and $f(N_{i}^{\prime })\text{=}f_{i,0}^{\prime }+f_{i,1}^{\prime }N_{i}^{\prime }+\cdots +$ $f_{i,n-1}^{\prime }N_{i}^{\prime n-1}$ are valid. If the above equations are valid, the roadside unit accepts vehicle data $m_{i}^{\prime }$. Otherwise, it outputs $\prime {⊥}^{\prime }$.

**Correctness:**

If the receiver receives the correct ciphertext, it can accurately decrypt the plaintext message. To prove that the scheme is correct, we only need to verify the correctness of the unsigncryption and the legitimacy of the aggregate signcryption ciphertext, as follows:

$$R_{i}^{\prime }=t_{i}\cdot P\cdot S\cdot H_{1}(ID_{i},PK_{i})\;=T_{i}^{\prime }\cdot SK_{i,2}\;$$ (2)

$$t_{i}x_{i}+R_{i}=t_{i}\cdot x_{i}+T_{i}\cdot S\cdot H_{1}(ID_{i},PK_{i,1})\;=T_{i}\cdot (x_{i}+S\cdot H_{1}(ID_{i},PK_{i}))\;=T_{i}\cdot (x_{i}+S\cdot PK_{i,3})\;=T_{i}\cdot SK_{i,2}\;=T_{i}\cdot tk_{j}$$ (3)

$$X_{agg}^{\prime }=\sum_{i=1}^{n}V_{i}^{\prime }\cdot P\;=\sum_{i=1}^{n}(d_{i}+h_{i}^{\prime }\cdot a_{i}+t_{i}\cdot h_{i}^{\prime \prime })\cdot P\;=\sum_{i=1}^{n}\left[r_{i}\cdot P+P_{pub}\cdot H_{1}(ID_{i},PK_{i})+h_{i}^{\prime }\cdot C_{i,1}+h_{i}^{\prime \prime }\cdot T_{i}\right]\;=\sum_{i=1}^{n}\left[M_{i}+P_{pub}\cdot H_{1}(ID_{i},PK_{i})+h_{i}^{\prime }\cdot C_{i,1}+h_{i}^{\prime \prime }\cdot T_{i}\right]\;=X_{agg}^{*}$$ (4)

## Security proof

### Confidentiality

**Theorem 1 (IND-CCA2-1).** If an adversary ${𝒜}_{1}$ wins the game with an undeniable advantage, the challenger 𝒞 can solve the CDH difficulty assumption within a finite polynomial time.

**Proof.** 𝒞 is the challenger to solve CDH difficult problems, ${𝒜}_{1}$ is an adversary. Given challenge example (*P*,*aP*,*bP*), $a,b\in Z_{q}^{*}$. 𝒞 can use ${𝒜}_{1}$ to calculate *abP*. The game is executed by 𝒞 and ${𝒜}_{1}$ is as follows:

1. **Setup: **𝒞 selects the challenge identity *ID*_*j*_, executes initialization algorithm, randomly selects $a\in Z_{q}^{*}$, calculates system public key *P*_*pub*_ = *aP*, and returns system parameters $para=\{p,q,P,P_{pub},H_{0},H_{1},H_{2},H_{3},H_{4},H_{5}\}$ and *ID*_*j*_ to $A_{1}$.
2. **Phase 1: **𝒞 needs to maintain eight initially empty lists *L*_*i*_(*i* = 0,1,2,3,4,5), *L*_*c*_, *L*_*x*_ to record the results of queries, *L*_1_ is also used for tracking key extraction queries. *L*_*c*_ is used for tracking test trapdoor queries. *L*_*x*_ is used for tracking secret value queries. The interactive process for 𝒞 and ${𝒜}_{1}$ is as follows.
  1) *H*_0_-queries: ${𝒜}_{1}$ submits the *H*_0_ query, 𝒞 searches $\left(ID_{i},H_{0}(ID_{i})\right)$ whether exists in list *L*_1_. If it exists, 𝒞 returns it to $A_{1}$. Otherwise, 𝒞 randomly selects $H_{0}(ID_{i})\in {𝑍}_{\text{q}}^{*}$ to return to $A_{1}$, and inserts $\left(ID_{i},PK_{i}\right)$ into *L*_1_.
  2) *H*_1_-queries: ${𝒜}_{1}$ submits the *H*_1_ query, 𝒞 searches $\left(ID_{i},H_{0}(ID_{i})\right)$ whether exists in list *L*_1_. If it exists, 𝒞 returns it to ${𝒜}_{1}$. Otherwise, 𝒞 randomly selects $H_{1}(ID_{i},PK_{i})\times G^{2}\in {𝑍}_{\text{q}}^{*}$ to return to ${𝒜}_{1}$, and inserts $\left(ID_{i},PK_{i}\right)$ into *L*_1_.
  3) *H*_2_-queries: ${𝒜}_{1}$ submits the *H*_2_ query, 𝒞 searches $(m_{i},ID_{i},ID_{j},T_{i},PK_{i},$ *PK*_*j*_) whether it exists in list *L*_2_. If it exists, 𝒞 returns it to ${𝒜}_{1}$. Otherwise, 𝒞 randomly selects $H_{2}(m_{i},ID_{i},ID_{j},T_{i},PK_{i},PK_{j})\in \{0,1{\}}^{*}$ to return to ${𝒜}_{1}$, and add $\left(m_{i},ID_{i},ID_{j},T_{i},PK_{i},PK_{j}\right)$ to *L*_2_.
  4) *H*_3_-queries: ${𝒜}_{1}$ submits the *H*_3_ query, 𝒞 searches $\left(m_{i},n,f_{i,0},\cdots ,f_{i,n-1}\right)$ whether exists in list *L*_3_. If it exists, 𝒞 returns it to ${𝒜}_{1}$. Otherwise, 𝒞 randomly selects $H_{3}(m_{i},n,f_{i,0},\cdots ,f_{i,n-1})\in Z_{q}^{*}$ to return to ${𝒜}_{1}$, and save it to *L*_3_.
  5) *H*_4_-queries: ${𝒜}_{1}$ submits the *H*_4_ query, 𝒞 searches $\left(R_{i},H_{4}(R_{i})\right)$ whether exists in list *L*_4_. If it exists, 𝒞 returns it to $A_{1}$. Otherwise, 𝒞 randomly selects $H_{4}(R_{i})\in Z_{q}^{*}$ to return to $A_{1}$, and save it to *L*_4_.
  6) *H*_5_-queries: ${𝒜}_{1}$ submits the *H*_5_ query, 𝒞 searches *C*_*i*,6_ whether exists in list *L*_5_. If it exists, 𝒞 returns it to ${𝒜}_{1}$. Otherwise, 𝒞 randomly selects $C_{i,6}\in {\;\;\;\{\;\;\text{ 0,1 }\;\;\}\;\;\;}^{l}$ to return to ${𝒜}_{1}$, and save it to *L*_5_.
  7) *td*-queries: ${𝒜}_{1}$ submits the *td* query. If $\left(ID_{i},PK_{i}\right)$ exists in *L*_1_, 𝒞 obtains *SK*_*j*,3_ through *H*_1_-queries, and returns $tk_{j}=SK_{j,3}$ to ${𝒜}_{1}$. Otherwise, 𝒞 randomly selects $tk_{j}\in Z_{q}^{*}$ to send to ${𝒜}_{1}$, and adds $\left(ID_{i},PK_{i}\right)$ to *L*_*c*_.
  8) Secret value queries: ${𝒜}_{1}$ performs the key query on identity *ID*_*i*_, 𝒞 query whether $x_{ID_{i}}$ exists in *L*_*x*_. If it exists in *L*_*x*_, returns $x_{ID_{i}}$ to ${𝒜}_{1}$. Otherwise, 𝒞 randomly selects $x_{ID_{i}}\in Z_{q}^{*}$ and adds $\left(ID_{i},x_{ID_{i}}\right)$ to *L*_*c*_.
  9) Public and private key queries: ${𝒜}_{1}$ performs the key query on identity *ID*_*i*_,𝒞 query whether $\left(ID_{i},PK_{i}\right)$ exists in *L*_1_, and if it exists, return it to ${𝒜}_{1}$. Otherwise, 𝒞 randomly selects $s_{i}\in {𝑍}_{\text{q}}^{*}$, calculates $PK_{i}=s_{i}P$, returns (*ID*_*i*_, *PK*_*i*_) to ${𝒜}_{1}$, and add $\left(ID_{i},s_{i},PK_{i}\right)$ to *L*_1_.
  10) Public key replacement queries: ${𝒜}_{1}$ performs the public key replacement query on $\left(X_{i}^{\prime },M_{i}^{\prime }\right)$, if $\left(ID_{i},X_{i}^{\prime },M_{i}^{\prime }\right)$ exists in *L*_1_, replace $\left(X_{i},M_{i}\right)$ with $\left(X_{i}^{\prime },M_{i}^{\prime }\right)$. Otherwise, add $\left(X_{i}^{\prime },M_{i}^{\prime }\right)$ to *L*_1_, set $x_{ID_{i}}$ to $\prime {⊥}^{\prime }$, and update the list *L*_*x*_.
  11) Aggregate signcryption queries: ${𝒜}_{1}$ selects $\left(ID_{i},m_{i},ID_{j}\right)$. 𝒞 query the list *L*_1_ to obtain the private key of the sender and the public key of the receiver. Then, the aggregate signcryption algorithm is executed to obtain the aggregated ciphertext $\sigma_{agg}=\left({\left\{C_{i}\right\}}_{i=1,2,\cdots ,n},X_{agg}\right)$.
  12) Aggregate unsigncryption queries: ${𝒜}_{1}$ selects $\left(ID_{i},\sigma_{agg},ID_{j}\right)$. 𝒞 performs validity verification. If the ciphertext is valid, 𝒞 executes the aggregate unsigncryption algorithm to obtain the set of plaintext messages $\{m_{i}{\}}_{i=1}^{n}$ and returns it to ${𝒜}_{1}$. Otherwise, $\prime {⊥}^{\prime }$ is returned.
3. **Challenge: **${𝒜}_{1}$ gives *ID*_*i*_ and *ID*_*j*_, along with two plaintext messages of equal length $\left(m_{0},m_{1}\right)$ to 𝒞. Upon receiving these inputs, 𝒞 randomly selects a sender identity $ID_{i}^{*}$ and a bit ξ∈{0,1}. If $ID_{i}^{*}$ is not equal to *ID*_*j*_, 𝒞 proceeds to run the signcryption algorithm on the plaintext message $m_{\xi }$, producing ciphertext *C*. Subsequently, 𝒞 employs the aggregate signcryption algorithm to obtain ${\text{C}}^{*}$, which is then returned to ${𝒜}_{1}$.
4. **Phase 2: **${𝒜}_{1}$ continues to execute the same inquiry as **Phase 1** after receiving ${\text{C}}^{*}$, but $A_{1}$ is unable to query the private key of $ID_{j}^{*}$ and cannot perform unsigncryption queries on ${\text{C}}^{*}$.
5. **Guess: **${𝒜}_{1}$ outputs a guess ξ for $\xi^{\prime }\in \{0,1\}$, if $\xi =\xi^{\prime }$, then ${𝒜}_{1}$ wins the above game. 𝒞 will use ${𝒜}_{1}$ to calculate *R*_*i*_ = *abP* as the solution of the CDH difficult problem.

Therefore, if an adversary ${𝒜}_{1}$ is able to break the confidentiality of the proposed scheme by successfully performing the above game, it means that the adversary has a nonnegligible advantage in breaking the CDHP. However, there is currently no effective solution to the difficult problem. Hence, our scheme satisfies the confidentiality under the first type of attack.

**Theorem 2 (IND-CCA2-2).** If an adversary ${𝒜}_{2}$ wins the game with an undeniable advantage, the challenger 𝒞 can solve the CDH difficulty assumption within a finite polynomial time.

**Proof.** 𝒞 is the challenger to solve CDH difficult problems, ${𝒜}_{2}$ is an adversary. Given challenge example (*P*,*aP*,*bP*), $a,b\in {𝑍}_{\text{q}}^{*}$. 𝒞 can use ${𝒜}_{2}$ to calculate *abP*. The game process for 𝒞 and ${𝒜}_{2}$ is as follows.

1. **Setup: **𝒞 selects challenge identity *ID*_*j*_, and returns system parameters *para* =  $\left\{p,q,P,\mathrm{\backslash break}P_{pub},H_{0},H_{1},H_{2},H_{3},H_{4},H_{5}\right\}$, *ID*_*j*_ and master key *s* to ${𝒜}_{2}$.
2. **Phase 1:** The interactive process for 𝒞 and ${𝒜}_{2}$ is as follows:
  1) *H*_0_-queries: ${𝒜}_{2}$ submits the *H*_0_ query, 𝒞 searches $\left(ID_{i},H_{0}(ID_{i})\right)$ whether exists in list *L*_1_. If it exists, C returns it to ${𝒜}_{1}$. Otherwise, 𝒞 randomly selects $H_{0}(ID_{i})\in {𝑍}_{\text{q}}^{*}$ to return to ${𝒜}_{2}$, and inserts $\left(ID_{i},PK_{i}\right)$ into *L*_1_.
  2) *H*_1_-queries: ${𝒜}_{2}$ submits the *H*_1_ query, 𝒞 searches $\left(ID_{i},H_{0}(ID_{i})\right)$ whether exists in list *L*_1_. If it exists, 𝒞 returns it to ${𝒜}_{1}$. Otherwise, 𝒞 randomly selects $H_{1}(ID_{i},PK_{i})\times G^{2}\in {𝑍}_{\text{q}}^{*}$ to return to ${𝒜}_{2}$, and inserts $\left(ID_{i},PK_{i}\right)$ into *L*_1_.
  3) *H*_2_-queries: ${𝒜}_{2}$ submits a *H*_2_ query, 𝒞 searches $\left(m_{i},ID_{i},ID_{j},T_{i},PK_{i},PK_{j}\right)$ whether it exists in list *L*_2_. If it exists, return it to ${𝒜}_{2}$. Otherwise, 𝒞 randomly selects $H_{2}(m_{i},ID_{i},ID_{j},T_{i},PK_{i},PK_{j})\in \{0,1{\}}^{*}$ to return to ${𝒜}_{1}$, and add $\left(m_{i},ID_{i},ID_{j},T_{i},PK_{i},PK_{j}\right)$ to *L*_2_.
  4) *H*_3_-queries: $A_{2}$ submits the *H*_3_ query, 𝒞 searches $\left(m_{i},n,f_{i,0},\cdots ,f_{i,n-1}\right)$ whether exists in list *L*_3_. If it exists, 𝒞 returns it to ${𝒜}_{2}$. Otherwise, C randomly selects $H_{3}(m_{i},n,f_{i,0},\cdots ,f_{i,n-1})\in {𝑍}_{q}^{*}$ to return to ${𝒜}_{2}$, and save it to *L*_3_.
  5) *H*_4_-queries: ${𝒜}_{2}$ submits the *H*_4_ query, 𝒞 searches $\left(R_{i},H_{4}(R_{i})\right)$ whether exists in list *L*_4_. If it exists, 𝒞 returns it to ${𝒜}_{2}$. Otherwise, 𝒞 randomly selects $H_{4}(R_{i})\in Z_{q}^{*}$ to return to ${𝒜}_{2}$, and save it to *L*_4_.
  6) *H*_5_-queries: ${𝒜}_{2}$ submits the *H*_5_ query, 𝒞 searches *C*_*i*,6_ whether exists in list *L*_5_. If it exists, 𝒞 returns it to ${𝒜}_{2}$. Otherwise, 𝒞 randomly selects $C_{i,6}\in {\;\;\;\{\;\;\text{ 0,1 }\;\;\}\;\;\;}^{l}$ to return to $A_{2}$, and save it to *L*_5_.
  7) *td*-queries: ${𝒜}_{2}$ submits a *td* query. If $\left(ID_{i},PK_{i}\right)$ exists in *L*_1_, 𝒞 obtains *SK*_*j*,3_ through *H*_1_-queries, and returns $tk_{j}=s_{k_{j,3}}$ to ${𝒜}_{2}$. Otherwise, 𝒞 randomly selects $tk_{j}\in Z_{q}^{*}$ to send to ${𝒜}_{2}$, and adds $\left(ID_{i},PK_{i}\right)$ to *L*_*c*_.
  8) Public and private key queries: $A_{2}$ perform a key query on identity *ID*_*i*_, 𝒞 query whether $\left(ID_{i},PK_{i}\right)$ exists in *L*_1_, and if it exists, return it to ${𝒜}_{2}$. Otherwise, C randomly selects $s_{i}\in {𝑍}_{\text{q}}^{*}$, calculates $PK_{i}=s_{i}P$, returns (*ID*_*i*_, *PK*_*i*_) to ${𝒜}_{2}$, and add $\left(ID_{i},s_{i},PK_{i}\right)$ to *L*_1_.
  9) Aggregate signcryption queries: ${𝒜}_{2}$ selects $\left(ID_{i},m_{i},ID_{j}\right)$. 𝒞 query the list *L*_1_ to obtain the private key of the sender and the public key of the receiver. Then, the aggregate signcryption algorithm is executed to obtain the aggregated ciphertext $\sigma_{agg}=\left({\left\{C_{i}\right\}}_{i=1,2,\cdots ,n},X_{agg}\right)$.
  10) Aggregate unsigncryption queries: ${𝒜}_{2}$ selects $\left(ID_{i},\sigma_{agg},ID_{j}\right)$. 𝒞 performs validity verification. If the ciphertext is valid, 𝒞 executes the aggregate unsigncryption algorithm to obtain the set of plaintext messages $\{m_{i}{\}}_{i=1}^{n}$ and returns it to ${𝒜}_{2}$. Otherwise, $\prime {⊥}^{\prime }$ is returned.
3. **Challenge: **${𝒜}_{2}$ gives *ID*_*i*_ and *ID*_*j*_, along with two plaintext messages of equal length $\left(m_{0},m_{1}\right)$ to C. Upon receiving these inputs, 𝒞 randomly selects a sender identity $ID_{i}^{*}$ and a bit ξ∈{0,1}. If $ID_{i}^{*}$ is not equal to *ID*_*j*_, 𝒞 proceeds to run the signcryption algorithm on the plaintext message $m_{\xi }$, producing ciphertext *C*. Subsequently, 𝒞 employs the aggregate signcryption algorithm to obtain ${\text{C}}^{*}$, which is then returned to ${𝒜}_{2}$.
4. **Phase 2: **${𝒜}_{2}$ continues to execute the same inquiry as **Phase 1** after receiving ${\text{C}}^{*}$, but ${𝒜}_{2}$ is unable to query the private key of $ID_{j}^{*}$ and cannot perform unsigncryption queries on ${\text{C}}^{*}$.
5. **Guess: **${𝒜}_{2}$ outputs a guess ξ for $\xi^{\prime }\in \{0,1\}$, if $\xi =\xi^{\prime }$, then ${𝒜}_{2}$ wins the above game. C will use ${𝒜}_{2}$ to calculate *R*_*i*_ = *abP* as the solution of the CDH difficult problem.

If adversary ${𝒜}_{2}$ can compromise the confidentiality of the proposed scheme by completing the described game, it implies that the adversary holds a significant advantage in breaking the CDHP. However, there is no feasible solution to solve this challenging problem yet. Therefore, the proposed scheme effectively ensures confidentiality against the first type of attack.

### Unforgeability

**Theorem 3 (EUF-CMA-1).** Given the ROM and the intractability of the CDHP problem, if there exists an adversary ${𝒜}_{1}$ who can win the EUF-CMA game in polynomial time, then the challenger C can resolve the difficult CDHP problem with a non-negligible advantage.

**Proof.** Challenger 𝒞 gives a random DLP instance (*P*,*aP*), $a\in {𝑍}_{\text{q}}^{*}$. The purpose of 𝒞 is to solve CDHP through interaction with opponent ${𝒜}_{1}$, that is, to find *a*.The game process for 𝒞 and ${𝒜}_{1}$ is as follows:

1. **Setup: **𝒞 sets *P*_*pub*_ = *aP*, and returns system parameters *para* = {*p*,*q*,*P*,*P*_*pub*_, $H_{0},H_{1},H_{2},H_{3},H_{4},H_{5}\}$ to ${𝒜}_{1}$.
2. **Queries: **$A_{1}$ adaptively performs hash query, public key query, private key query, public key replacement query and signcryption query. 𝒞 needs to maintain eight initially empty lists *L*_*i*_(*i* = 0,1,2,3,4,5), *L*_*c*_, *L*_*x*_ to record the results of queries. *L*_1_ is also used for tracking key extraction queries. *L*_*c*_ is used for tracking test trapdoor queries. *L*_*x*_ is used for tracking secret value queries. The interactive process for 𝒞 and ${𝒜}_{1}$ is as follows.
  1) *H*_0_-queries: ${𝒜}_{1}$ submits the *H*_0_ query, 𝒞 searches $\left(ID_{i},H_{0}(ID_{i})\right)$ whether exists in list *L*_1_. If it exists, 𝒞 returns it to ${𝒜}_{1}$. Otherwise, 𝒞 randomly selects $H_{0}(ID_{i})\in {𝑍}_{\text{q}}^{*}$ to return to ${𝒜}_{1}$, and inserts $\left(ID_{i},PK_{i}\right)$ into *L*_1_.
  2) *H*_1_-queries: ${𝒜}_{1}$ submits the *H*_1_ query, 𝒞 searches $\left(ID_{i},H_{0}(ID_{i})\right)$ whether exists in list *L*_1_. If it exists, 𝒞 returns it to ${𝒜}_{1}$. Otherwise, 𝒞 randomly selects $H_{1}(ID_{i},PK_{i})\times G^{2}\in {𝑍}_{\text{q}}^{*}$ to return to ${𝒜}_{1}$, and inserts $\left(ID_{i},PK_{i}\right)$ into *L*_1_.
  3) *H*_2_-queries: ${𝒜}_{1}$ submits a *H*_2_ query, 𝒞 searches $\left(m_{i},ID_{i},ID_{j},T_{i},PK_{i},PK_{j}\right)$ whether it exists in list *L*_2_. If it exists, 𝒞 returns it to ${𝒜}_{1}$. Otherwise, 𝒞 randomly selects $H_{2}(m_{i},ID_{i},ID_{j},T_{i},PK_{i},PK_{j})\in \{0,1{\}}^{*}$ to return to ${𝒜}_{1}$, and adds (*m*_*i*_,
    $ID_{i},ID_{j},T_{i},PK_{i},PK_{j})$ to *L*_2_.
  4) *H*_3_-queries: ${𝒜}_{1}$ submits the *H*_3_ query, 𝒞 searches $\left(m_{i},n,f_{i,0},\cdots ,f_{i,n-1}\right)$ whether exists in list *L*_3_. If it exists, 𝒞 returns it to ${𝒜}_{1}$. Otherwise, 𝒞 randomly selects $H_{3}(m_{i},n,f_{i,0},\cdots ,f_{i,n-1})\in Z_{q}^{*}$ to return to ${𝒜}_{1}$, and adds it to *L*_3_.
  5) *H*_4_-queries: ${𝒜}_{1}$ submits the *H*_4_ query, 𝒞 searches $\left(R_{i},H_{4}(R_{i})\right)$ whether exists in list *L*_4_. If it exists, return it to ${𝒜}_{1}$. Otherwise, 𝒞 randomly selects $H_{4}(R_{i})\in Z_{q}^{*}$ to return to ${𝒜}_{1}$, and save it to *L*_4_.
  6) *H*_5_-queries: ${𝒜}_{1}$ submits the *H*_5_ query, 𝒞 searches *C*_*i*,6_ whether exists in list *L*_5_. If it exists, 𝒞 returns it to ${𝒜}_{1}$. Otherwise, 𝒞 randomly selects $C_{i,6}\in {\;\;\;\{\;\;\text{ 0,1 }\;\;\}\;\;\;}^{l}$ to return to ${𝒜}_{1}$, and save it to *L*_5_.
  7) *td*-queries: ${𝒜}_{1}$ submits a *td* query. If $\left(ID_{i},PK_{i}\right)$ exists in *L*_1_, 𝒞 obtains *SK*_*j*,3_ through *H*_1_-queries, and returns $tk_{j}=SK_{j,3}$ to ${𝒜}_{1}$. Otherwise, 𝒞 randomly selects $tk_{j}\in Z_{q}^{*}$ to send to ${𝒜}_{1}$, and adds $\left(ID_{i},PK_{i}\right)$ to *L*_*c*_.
  8) Secret value queries: ${𝒜}_{1}$ performs a key query on identity *ID*_*i*_, *C* query whether $x_{ID_{i}}$ exists in *L*_*x*_. If it exists in *L*_*x*_, returns $x_{ID_{i}}$ to ${𝒜}_{1}$. Otherwise, C randomly selects $x_{ID_{i}}\in Z_{q}^{*}$ and adds $\left(ID_{i},x_{ID_{i}}\right)$ to *L*_*c*_.
  9) Public and private key queries: ${𝒜}_{1}$ performs a key query on identity *ID*_*i*_, *C* query whether $\left(ID_{i},PK_{i}\right)$ exists in *L*_1_, and if it exists, return it to ${𝒜}_{1}$. Otherwise, 𝒞 randomly selects $s_{i}\in {𝑍}_{\text{q}}^{*}$, calculates $PK_{i}=s_{i}P$, returns $\left(ID_{i},PK_{i}\right)$ to ${𝒜}_{1}$, and add $\left(ID_{i},s_{i},PK_{i}\right)$ to *L*_1_.
  10) Public key replacement queries: ${𝒜}_{1}$ performs a public key replacement query on $\left(X_{i}^{\prime },M_{i}^{\prime }\right)$ , if $\left(ID_{i},X_{i}^{\prime },M_{i}^{\prime }\right)$ exists in *L*_1_, replace $\left(X_{i},M_{i}\right)$ with $\left(X_{i}^{\prime },M_{i}^{\prime }\right)$. Else, 𝒞 add $\left(X_{i}^{\prime },M_{i}^{\prime }\right)$ to *L*_1_, set $x_{ID_{i}}$ to $\prime {⊥}^{\prime }$, and update the list *L*_*x*_.
  11) Aggregate signcryption queries: ${𝒜}_{1}$ selects $\left(ID_{i},m_{i},ID_{j}\right)$. 𝒞 queries the list *L*_1_ to obtain the private key of the sender and the public key of the receiver. In addition, the aggregate signcryption algorithm is executed to obtain the aggregated ciphertext $\sigma_{agg}=\left({\left\{C_{i}\right\}}_{i=1,2,\cdots ,n},X_{agg}\right)$.
  12) Aggregate unsigncryption queries: ${𝒜}_{1}$ selects $\left(ID_{i},\sigma_{agg},ID_{j}\right)$. 𝒞 performs validity verification. If the ciphertext is valid, 𝒞 executes the aggregate unsigncryption algorithm to obtain the set of plaintext messages $\{m_{i}{\}}_{i=1}^{n}$ and returns it to ${𝒜}_{1}$. Otherwise, $\prime {⊥}^{\prime }$ is returned.

**Forgery phase:** After the inquiry phase, forge and output *n* signers $ID_{i}^{*}$ to create an aggregate signature ciphertext $\sigma_{agg}^{*}=\left({\left\{C_{i}^{*}\right\}}_{i=1,2,\cdots ,n},X_{agg}^{*}\right)$for the message $m_{i}^{*}$. Assuming that at least one out of the *n* users, specifically user *D*_1_, has not been queried for their secret value. If the forged aggregate signature is valid, then

$$X_{agg}^{*}=\sum_{i=1}^{n}\left[M_{i}+P_{pub}H_{1}(ID_{i},PK_{i})+h_{i}^{\prime }C_{i,1}+h_{i}^{\prime \prime }T_{i}\right]\;=\sum_{i=1}^{n}\left[r_{i}P+P_{pub}H_{1}(ID_{i},PK_{i})+h_{i}^{\prime }C_{i,1}+h_{i}^{\prime \prime }T_{i}\right]\;=\sum_{i=1}^{n}(d_{i}+h_{i}^{\prime }a_{i}+t_{i}h_{i}^{\prime \prime })P\;=\sum_{i=1}^{n}(d_{i}+t_{i}h_{i}^{\prime \prime })P+\sum_{i=2}^{n}(h_{i}^{\prime }a_{i})P+h_{1}^{\prime }aP$$ (5)

If 𝒞 can calculate $a={\left(h_{1}^{\prime }P\right)}^{-1}\left(X_{agg}^{*}-\sum_{i=1}^{n}\left(d_{i}+t_{i}h_{i}^{\prime \prime }\right)P-\sum_{i=2}^{n}\left(h_{i}^{\prime }a_{i}\right)P\right)$, then 𝒞 has successfully solved the CDHP problem. The advantage of the ${𝒜}_{1}$ to win the game is negligible, thus the premise for 𝒞 to successfully solve the CDHP does not exist. According to definition 3, the scheme satisfies the EUF-CMA.

**Theorem 4 (EUF-CMA-2).** Given the ROM and the intractability of the CDHP problem, if there exists an adversary $A_{2}$ who can win the EUF-CMA game in polynomial time, then the challenger 𝒞 can resolve the difficult CDHP problem with a non-negligible advantage.

**Proof.** Challenger 𝒞 gives a random DLP instance (*P*,*aP*), $a\in {𝑍}_{\text{q}}^{*}$. The purpose of 𝒞 is to solve CDHP through interaction with opponent ${𝒜}_{2}$, that is, to find *a*. The game process for 𝒞 and ${𝒜}_{2}$ is as follows.

1. **Setup: **𝒞 sets *P*_*pub*_ = *aP*, and returns system parameters *para* = {*p*,*q*,*P*,*P*_*pub*_, $H_{0},H_{1},H_{2},H_{3},H_{4},H_{5}\}$ and master key *s* to ${𝒜}_{2}$.
2. **Queries:** In addition to mastering the given conditions in theorem 3, ${𝒜}_{2}$ also owns the system master key *s*. ${𝒜}_{2}$ can perform all queries except “public key replacement query” in theorem 3.

**Forgery phase:** After the inquiry phase, forge and output *n* signers $ID_{i}^{*}$ to create an aggregate signature ciphertext $\sigma_{agg}^{*}=\left({\left\{C_{i}^{*}\right\}}_{i=1,2,\cdots ,n},X_{agg}^{*}\right)$for the message $m_{i}^{*}$. Assuming that at least one out of the *n*users, specifically user *D*_1_, has not been queried for their secret value.

If 𝒞 can calculate $a={\left(h_{1}^{\prime }P\right)}^{-1}\left(X_{agg}^{*}-\sum_{i=1}^{n}(d_{i}+t_{i}h_{i}^{\prime \prime })P-\sum_{i=2}^{n}(h_{i}^{\prime }a_{i})P\right)$ by calculating


$$X_{agg}^{*}=\sum_{i=1}^{n}\left[M_{i}+P_{pub}H_{1}(ID_{i},PK_{i})+h_{i}^{\prime }C_{i,1}+h_{i}^{\prime \prime }T_{i}\right]$$

$$=\sum_{i=1}^{n}(d_{i}+t_{i}h_{i}^{\prime \prime })P+\sum_{i=2}^{n}(h_{i}^{\prime }a_{i})P+h_{1}^{\prime }aP,$$

then 𝒞 has successfully solved the CDHP problem. The advantage of ${𝒜}_{2}$ to win the game is negligible, thus the premise for 𝒞 to successfully solve the CDHP does not exist. According to definition 4, the scheme satisfies the EUF-CMA.

## Security analysis

### Confidentiality of data

In the IoV, vehicular data is safeguarded through key encryption prior to transmission. For instance, *OBU*_*i*_ computes the ciphertext of the message *m*_*i*_. The key is derived through the hash function. If an adversary intends to obtain a random value *x*_*i*_, it must first recover *X*_*i*_ based on the private key of the *OBU*_*i*_, and then perform the calculation to obtain *x*_*i*_.

However, the computational complexity of CDHP makes it infeasible for an adversary to calculate *X*_*i*_. As a result, messages containing vehicle data in the IoV will remain secure and ensure data confidentiality. The relevant security proof has been provided in **Confidentiality**.

### Unforgeability of data

In the signcryption phase, *OBU*_*i*_ outputs the ciphertexts $C_{i,1}=a_{i}P$, $C_{i,2}=b_{i}P$, $C_{i,3}=V_{i}P$, $C_{i,4}=R_{i}⊕(m_{i}∥V_{i})$, $C_{i,5}=H_{4}(t_{i}X_{i}+R_{i})⊕\left(N_{i}\|f(N_{i})\right)$, $C_{i,6}=H_{5}(n\|C_{i,1}\|\cdots \|C_{i,5}\|b_{i}PK_{j,2}\|f_{i,0}\|\cdots \|f_{i,n-1})$, of which $T_{i}=t_{i}P$, $R_{i}=t_{i}P_{pub}H_{1}(ID_{i},PK_{i})$, $V_{i}=d_{i}+h_{i}a_{i}+t_{i}h_{i}^{\prime }$, $X_{i}=x_{i}P$. *d*_*i*_, *r*_*i*_, *t*_*i*_, and *x*_*i*_ are the secret numbers randomly selected by *OBU*_*i*_, and $\left(d_{i},M_{i}\right)$ is the private key of *OBU*_*i*_. The adversary cannot obtain the secret number and private key, so it cannot recover the plaintext message or forge the legitimate ciphertext.

The encrypted ciphertext is sent by *OBU*_*i*_ to *RSU*_*i*_. The *RSU*_*i*_ decrypts ciphertext and obtains message *m*, then verify the decryption process. If the result is yes, this indicates that the vehicle data has not been changed during transmission. That is, unforgeability is achieved under the condition of CDHP difficult problems.

The relevant security proof has been provided in **Unforgeability**.

### Side channel attack defenses

The proposed scheme utilizes certificateless aggregate signcryption to significantly reduce computational overhead while enhancing security. By leveraging the aggregation feature, it lowers the risk of individual message attacks and complicates potential threats. Additionally, the multi-ciphertext equality test technology minimizes sensitivity to computational overhead, allowing for efficient processing of multiple ciphertexts, which balances power consumption and execution time. This dual approach not only improves efficiency but also provides strong protection against side channel attacks.

Our scheme effectively combines efficiency and enhanced security, making it suitable for secure communication in IoV vulnerable to side channel effects.

### Man-in-the-middle attack defense

The proposed scheme uses certificateless technology, which uses KGC, which eliminates the need for PKI. The proposed scheme uses certificateless technology, which uses KGC, which eliminates the need for PKI. This simplification reduces the complexity of certificate management and reduces the risk of man-in-the-middle attack ( MITI ) due to forged certificates.Moreover, the integration of certificateless aggregate signcryption enhances security by combining digital signatures and encryption, ensuring message integrity. Additionally, multi-ciphertext equality test technology allows for verification of message consistency, ensuring that the received data matches what was originally sent.

By combining the above technologies, this method not only protects the communic- ation channel, but also improves the security of information.

## Efficiency and performance analysis

In this section, we will conduct a comprehensive analysis and comparison of the security and computational efficiency performance between our proposed scheme and relevant certificateless signcryption schemes. Specific performance comparison results will be provided to illustrate the differences. The computational complexity of bilinear mapping is quite high. The certificateless signcryption schemes that do not use bilinear mapping have significant efficiency advantages over existing schemes that do.

As a result, the proposed scheme offers considerable benefits in terms of efficiency and security when compared to certificateless signcryption schemes that use bilinear mapping. In comparison to certificateless signcryption schemes that do not use bilinear mapping [16,18], the computational overhead mainly depends on the complexity of the signcryption and signcryption verification algorithms. It is primarily determined by counting the number of point multiplication and exponential operations performed on the group.

In order to evaluate the performance of the solution more comprehensively, the simulation was conducted on a host with the Intel (R) Xeon (R) Gold 6133 CPU @ 2.50GHz CPU and 8.0GB of memory, using PBC cryptography library on Ubuntu Server 22.04 LTS operating system. The computational overhead is shown in Table 1. For ease of representation, *T*_1_ represents scalar multiplication operations on a group *G*, *T*_2_ represents power operations on a group *G*, *P* represents bilinear pairing operations, and *n* represents the number of messages/recipients.

**Table 1:** Basic operation computational overhead.

Operation	*T*_1_	*T*_2_	*P*
Time(ms)	0.13	1.882	16.064

In this paper, we conduct a comparative analysis between the proposed scheme and the approaches presented in references [16,18,27,28] with regards to computational overhead. The findings of this analysis can be found in Table 2.

**Table 2:** Basic operation computational overhead.

Scheme	Signcryption	Unsigncryption
[16]	6*nT*_1_ + *nP*	*nT*_1_ + *nP*
[18]	$6nT_{1}+2nP+2nT_{2}$	3*nT*_1_ + 5*nP*
[27]	$2nT_{1}+6nT_{2}$	4*nP* + 2*nT*_2_
[28]	$2nT_{1}+6nT_{2}$	*n*(3*n* + 1)*T*_1_
Ours	7*nT*_1_	*n*(2 + 4*n*)*T*_1_

The proposed scheme uses multi-ciphertext equality test technology to support multi-user ciphertext retrieval, and supports testers to retrieve multiple ciphertexts at the same time, which improves the efficiency of ciphertext retrieval in multi-user environment. In addition, the scheme improves the efficiency of verifying user ciphertext in multi user environment by introducing aggregate signcryption.

**Fig 2:**
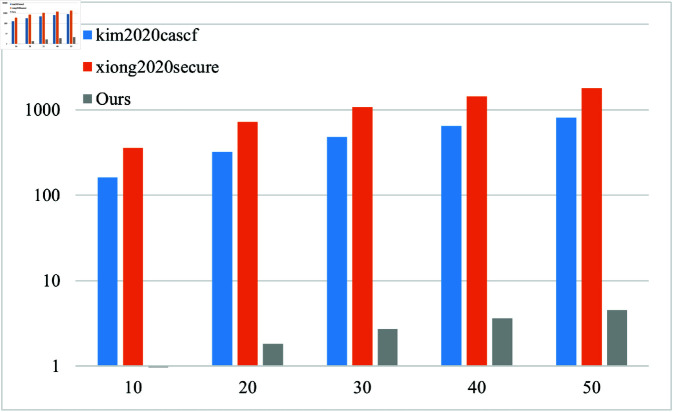
Computational overhead at signcryption phase.

**Fig 3:**
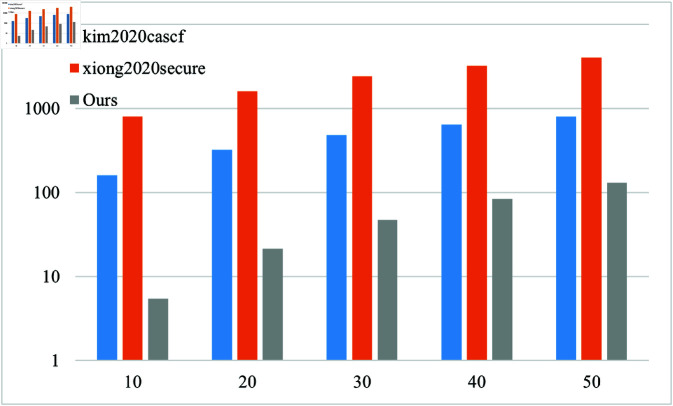
Computational overhead at unsigncryption phase.

The analysis results show that compared with similar schemes, the proposed scheme has lower computational overhead in the phases of ciphertext generation, ciphertext equality test, and ciphertext verification in a multi user environment. Signcryption and unsigncryption computational overhead are shown in Fig 2 and Fig 3.

In this paper, we compare the proposed scheme with the methods proposed in references [16,18,27,28] in terms of feature. The analysis results are shown in Table 3.

**Table 3:** Feature comparison.

Scheme	Certificateless Cryptography	Multi-ciphertext Equality Test	Confidentiality	Unforgeability
[16]	✓	×	✓	✓
[18]	×	×	✓	×
[27]	×	✓	✓	✓
[28]	×	✓	✓	✓
Ours	✓	✓	✓	

Compared with references [16,18,27,28], the proposed scheme uses multi-ciphertext equality test to retrieve multiple ciphertexts in the IoV at the same time. Compared with [18], the scheme introduces the certificateless aggregate signcryption technology, eliminates the key management problem, and proves that the scheme meets the unforgeability under the CDHP assumption.

## Conclusion

Certificateless aggregate signcryption combines the benefits of certificateless cryptography, allowing for efficient transmission and verification of signcryption data, and reducing the computational and communication overhead significantly. Aiming at the problems of low computational efficiency and data security of existing IoV cryptosystems in multi user environments,we proposes an aggregate signcryption scheme for IoV that supports multi-ciphertext equality test. The utilization of a certificateless cryptosystem eradicates the additional burden of certificate administration that is typically associated with conventional public key approaches.

The introduction of multi-ciphertext equality test technology enables multiple data users to simultaneously retrieve multiple vehicle data ciphertext, reducing the computational overhead of ciphertext equality test in a multi user environment. The use of aggregate signcryption technology improves the efficiency of signcryption the data of multiple vehicle users. the proposed scheme satisfies the confidentiality of vehicle data during transmission. The comparation analysis of the proposed scheme with similar approaches reveals that it offers better security with reduced computational overhead.

Looking forward to the future, the proposed aggregate signcryption scheme can be further enhanced by post-quantum cryptography to solve the future challenges brought by quantum computing. In addition, further optimization can bring more efficient processing and lower computational overhead, making the scheme more suitable for high-demand IoV environments.
